# P-1317. Antimicrobial Activities of Aztreonam-Avibactam and Comparator Agents Tested against Enterobacterales from European Hospitals Analysed by Infection Type (2022-2024)

**DOI:** 10.1093/ofid/ofaf695.1505

**Published:** 2026-01-11

**Authors:** Helio SaderJohn Kimbrough, Gregory Stone, Katherine Perez, Rodrigo E Mendes, Mariana Castanheira

**Affiliations:** Element Iowa City (JMI Laboratories), North Liberty, Iowa; Pfizer, Inc., Groton, Connecticut; Pfizer, Inc., Groton, Connecticut; Element Iowa City (JMI Laboratories), North Liberty, Iowa; Element, North Liberty, IA

## Abstract

**Background:**

Aztreonam-avibactam (ATM-AVI) was approved for clinical use in the European Union in April 2024 and in the United States in February 2025. We evaluated the activity of ATM-AVI and comparators against Enterobacterales (ENT) from Europe (EU).

**Table 1.**
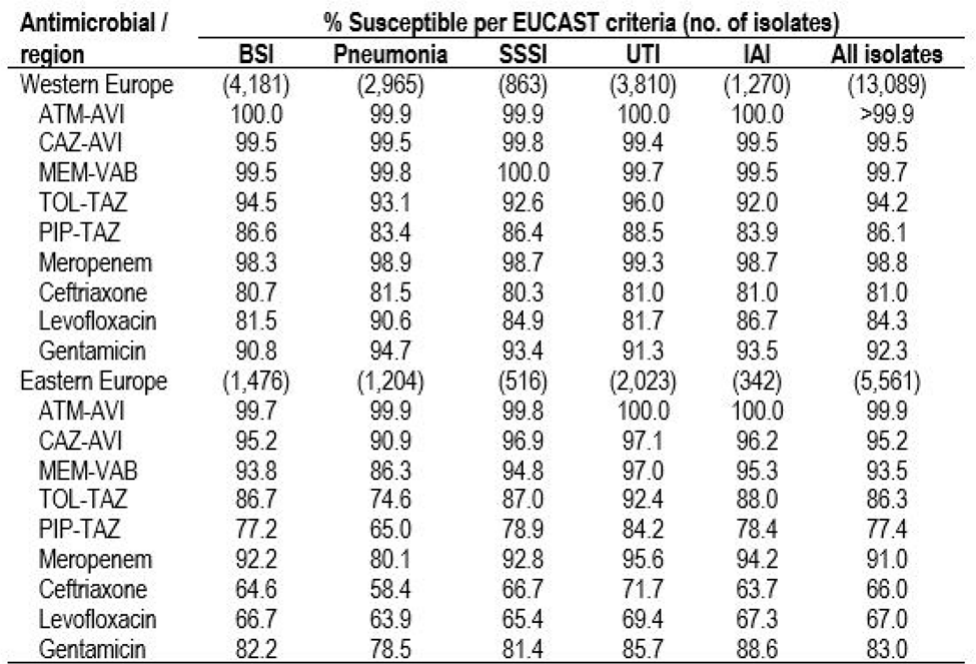
Antimicrobial susceptibility of Enterobacterales stratified by European region and infection type. Abbreviations: BSI, bloodstream infection; SSSI, skin and skin structure infection; UTI, urinary tract infection; IAI, intra-abdominal infection; ATM-AVI, aztreonam-avibactam; CAZ-AVI, ceftazidime-avibactam, MEM-VAB, meropenem-vaborbactam; TOL-TAZ, ceftolozane-tazobactam; PIP-TAZ, piperacillin-tazobactam.

**Figure:**
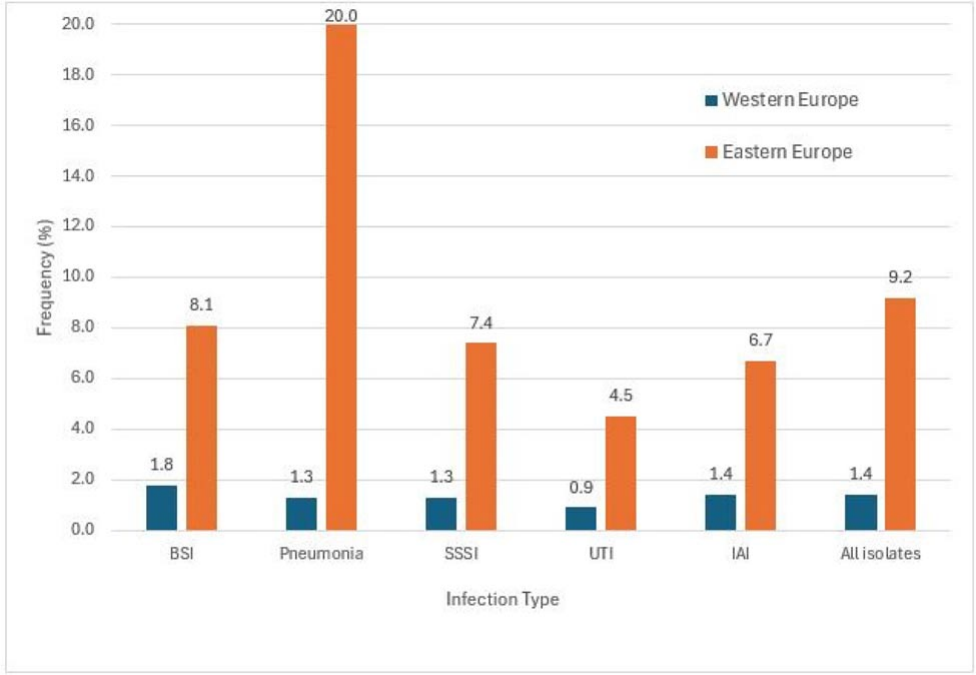
Frequency of carbapenem-resistant Enterobacterales (CRE) stratified by European region and infection type Abbreviations: BSI, bloodstream infection; SSSI, skin and skin structure infection; UTI, urinary tract infection; IAI, intra-abdominal infection.

**Methods:**

18,650 isolates were consecutively collected from 40 medical centers, 25 from Western EU (W-EU; n=13,089; 10 countries) and 15 from Eastern EU (E-EU; n=5,561; 9 countries). Isolates were susceptibility tested by broth microdilution methods in a central laboratory. The antimicrobial susceptibility and frequency of key resistance phenotypes were assessed and stratified by infection type: bloodstream (BSI; 5,657 isolates; 30.3%), pneumonia (4,169; 22.4%), skin and skin structure (SSSI; 1,379; 7.4%), urinary tract (UTI; 5,833; 31.3%), and intra-abdominal (1,612; 8.6%). EUCAST breakpoints were applied. Carbapenem-resistant ENT (CRE) were screened for carbapenemases (CBase) by whole genome sequencing.

**Figure:**
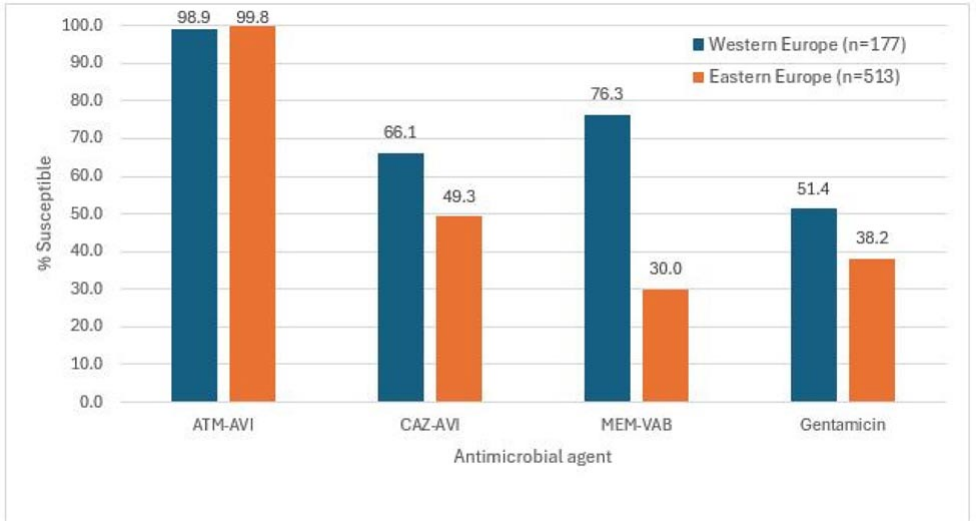
Antimicrobial susceptibility of carbapenem-resistant Enterobacterales (CRE) stratified by European region. Abbreviations: ATM-AVI, aztreonam-avibactam; CAZ-AVI, ceftazidime-avibactam, MEM-VAB, meropenem-vaborbactam.

**Figure:**
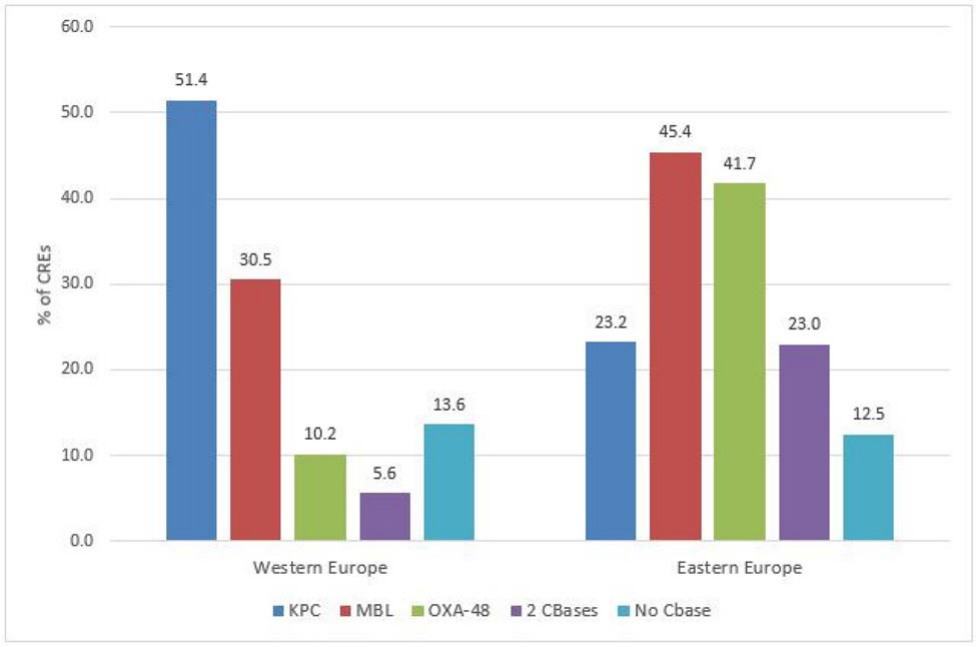
Distribution of carbapenemase (CBase) types stratified by European region.

**Results:**

ATM-AVI was active against 99.9-100.0% of W-EU isolates (MIC_50/90_, ≤ 0.03/0.12 mg/L) and 99.7-100.0% of E-EU isolates (MIC_50/90_, ≤ 0.03/0.25 mg/L). Resistance to comparator agents (Table 1) as well as the frequencies of CRE (Figure 1), multidrug-resistant, extensive-drug resistant, and difficult-to-treat resistant isolates were markedly higher among isolates from E-EU compared to W-EU for all infection types. ATM-AVI was active against 98.9% of CRE from W-EU and 99.8% from E-EU (MIC_50/90_, 0.25/0.5 mg/L in both regions). All comparators exhibited limited activity against CRE, including ceftazidime-avibactam (CAZ-AVI; 66.1% susceptible [S] in W-EU and 49.3% S in E-EU) and meropenem-vaborbactam (MEM-VAB; 76.3% S in W-EU and 30.0% S in E-EU; Figure 2). A CBase was identified in 153 CREs from W-EU (86.4%) and 513 CREs from E-EU (87.5%). The frequencies of CBase types varied markedly by EU region; the most common CBase types were KPC (51.4% of CREs) and NDM (18.6%) in W-EU and NDM (43.3%) and OXA-48 (41.7%) in E-EU (Figure 3).

**Conclusion:**

ATM-AVI demonstrated potent activity against Enterobacterales, including CREs, from all infection types in W-EU and E-EU. The activities of CAZ-AVI and MEM-VAB were compromised by the elevated frequencies of MBLs and OXA-48 types, especially in E-EU.

**Disclosures:**

Helio Sader, United States Food and Drug Administration: FDA Contract Number: 75F40123C00140 Katherine Perez, PhD, Pfizer: Stocks/Bonds (Public Company) Rodrigo E. Mendes, PhD, GSK: Grant/Research Support|Shionogi & Co., Ltd.: Grant/Research Support|United States Food and Drug Administration: FDA Contract Number: 75F40123C00140 Mariana Castanheira, PhD, Melinta Therapeutics: Advisor/Consultant|Melinta Therapeutics: Grant/Research Support

